# Periodic Mesoporous Organosilica Functionalized with Sulfonic Acid Groups as Acid Catalyst for Glycerol Acetylation

**DOI:** 10.3390/ma6083556

**Published:** 2013-08-16

**Authors:** Els De Canck, Inmaculada Dosuna-Rodríguez, Eric M. Gaigneaux, Pascal Van Der Voort

**Affiliations:** 1 Center for Ordered Materials, Organometallics & Catalysis (COMOC), Department of Inorganic and Physical Chemistry, Ghent University, Krijgslaan 281, Building S3, Ghent B-9000, Belgium; E-Mail: els.decanck@ugent.be; 2 Institute of Condensed Matter and Nanosciences (IMCN)—Division “MOlecules, Solids and reactiviTy—MOST”, Université catholique de Louvain, Croix du Sud 2/L7.05.17, Louvain-la-Neuve B-1348, Belgium; E-Mails: dosuna.inma@gmail.com (I.D.R.); eric.gaigneaux@uclouvain.be (E.M.G.)

**Keywords:** Periodic Mesoporous Organosilica, sulfonic acid groups, catalysis, glycerol acetylation

## Abstract

A Periodic Mesoporous Organosilica (PMO) functionalized with sulfonic acid groups has been successfully synthesized via a sequence of post-synthetic modification steps of a *trans*-ethenylene bridged PMO material. The double bond is functionalized via a bromination and subsequent substitution obtaining a thiol functionality. This is followed by an oxidation towards a sulfonic acid group. After full characterization, the solid acid catalyst is used in the acetylation of glycerol. The catalytic reactivity and reusability of the sulfonic acid modified PMO material is investigated. The catalyst showed a catalytic activity and kinetics that are comparable with the commercially available resin, Amberlyst-15, and furthermore our catalyst can be recycled for several subsequent catalytic runs and retains its catalytic activity.

## 1. Introduction

The discovery of Periodic Mesoporous Organosilicas (PMOs) [1,2,3] with organic bridging groups incorporated in their silica framework has been the start of a fascinating research area which provides materials with huge potential [4,5,6]. Different organic bridges have been employed for very diverse applications, such as heterogeneous catalysts [7,8], bio-sensors [9,10], chromatographic packing materials [11,12], low-k materials [13,14], adsorbents of pollutants [15] and controlled drug delivery systems [16,17,18,19]. PMOs are highly porous materials with large specific surface areas, pore volumes and narrow pore size distributions. Furthermore, they exhibit a high thermal and mechanical stability [20,21,22], especially in comparison with other porous silica materials [23]. This type of material is synthesized with structure directing agents such as the non-ionic triblock copolymer P123. Around this template, a silica source is condensed in basic or acid aqueous environment. Usually, an organo bis-silane (R′O)_3_–Si–R–Si–(OR′)_3_ is used where R represents the organic bridging group and R′ usually a methyl or ethyl group. Already many reports have appeared on different bridging groups (R) like phenylene, ethylene, ethenylene and ethylbenzene but also more complex and flexible organic functionalities have been described. Furthermore the bridging group can be modified to fine-tune the material for a specific application such as solid acid catalysis [4].

Concerning this topic, some very promising results have already been published regarding the incorporation of an acid functionality such as a sulfonic acid group and its catalytic activity. Several diverse methods have been applied to prepare sulfonic acid containing PMO materials. These strategies include the direct sulfonation of the phenylene bridge [24,25,26], as first attempted by Inagaki* et al.* [27], and the cocondensation of an organo bis-silane with (3-mercaptopropyl) trimethoxysilane (MPTMS) [28,29,30,31] followed by an oxidation of the thiol functionality. The latter can also be achieved by the *in-situ* oxidation of the thiol functionality by the addition of H_2_O_2_ during the cocondensation process of tetraethoxyorthosilicate (TEOS) and MPTMS [32,33]. Other silanes have been used in cocondensation processes with an organo bis-silane such as 2-(4-chlorosulfonylphenyl)-trimethoxysilane [34] and perfluorinated alkylsulfonic acid silanes [35,36,37].

In the specific case of ethenylene bridged PMOs, –SO_3_H moieties can be acquired by the direct sulfonation of the C=C bond [38]. However, the sulfonic acid group can detach from the material, depending on the environment used during catalysis. Another explored route is the use of a Diels Alder reaction where the ethene bond acts as dienophile. Kondo* et al.* [39,40] described the cycloaddition of the ethene bond with benzocyclobutene and subsequently the resulting phenylene moiety is sulfonated to obtain a heterogeneous catalyst. These authors tested this material for several catalytic reactions (esterification of acetic acid with ethanol, the Beckmann and pinacole-pinacolone rearrangement) and the catalyst showed excellent conversion results. This Diels Alder process has been further used to expand the functionalization possibilities [41].

Another example of modifying the surface of an ethenylene bridged PMO material has been reported by the research group of Kaliaguine [42]. First, the surface silanols were end-capped with hexamethyldisilazane after which a Friedel-Crafts alkylation with benzene was made, and subsequently the benzene moiety was sulfonated with concentrated sulfuric acid. The –SO_3_H containing material exhibited a high catalytic activity in the self-condensation of heptanal.

In this study, the pure *trans*-ethenylene bridged PMO material [43] is chosen as support material. Thiol functionalities were incorporated according to a procedure previously described by our research group [44] and subsequently oxidized in order to obtain –SO_3_H. This material was thoroughly characterized and the solid acid was tested in the acetylation of glycerol. Furthermore, the reusability of this catalyst was investigated.

## 2. Results and Discussion

**Figure 1:**
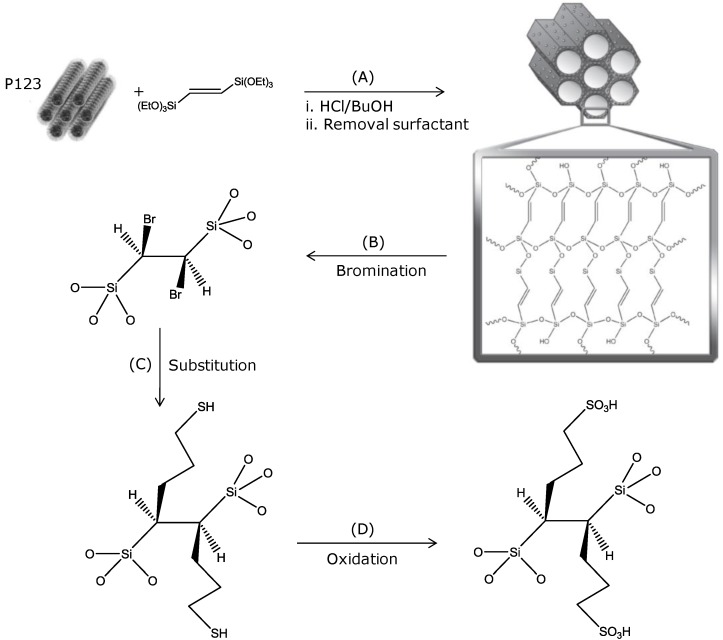
Summary of the synthetic pathways followed in this study. (**A**) Preparation of *trans*-ethenylene bridged Periodic Mesoporous Organosilica [EP]; (**B**) Bromination of EP [BEP]; (**C**) Substitution of the bromine with Grignard reagent of 3-chloro-1-propanethiol [EP–(CH_2_)_3_–SH] and (**D**) Oxidation with sulfuric acid [EP–(CH_2_)_3_–SO_3_H].

Starting from the *trans*-ethenylene bridged PMO (EP), a sulfonic acid modified PMO material is prepared, characterized and its catalytic activity is studied in the acetylation of glycerol. A general overview of the preparation method, including the starting material, is presented in Figure 1. Firstly, an ethenylene bridged Periodic Mesoporous Organosilica is synthesized starting from E-1,2-bis(triethoxysilyl)ethene (Figure 1, pathway A) [43,45].

Afterwards, the ethene bridge can be further functionalized to incorporate sulfonic acid groups in the material. When pathway B is followed, the material EP is firstly brominated and subsequently the bromine can be substituted via an SN2 reaction by the Grignard reagent of 3-chloro-1-propanethiol (Figure 1, pathway C). A material with a propylthiol group is obtained (EP–(CH_2_)_3_–SH) [44,46]. Oxidizing the thiol moiety is performed with H_2_SO_4_ or other oxidizing agents such as HNO_3_ [28,29,30,31] or H_2_O_2_ [25,47,48]. In this study, a treatment of EP–(CH_2_)_3_–SH with sulfuric acid and a thorough washing step was selected by which it converted the –SH into a –SO_3_H group (Figure 1, pathway D). This route resulted in the material EP–(CH_2_)_3_–SO_3_H.

### 2.1. Characterization of the Solids

**Figure 2:**
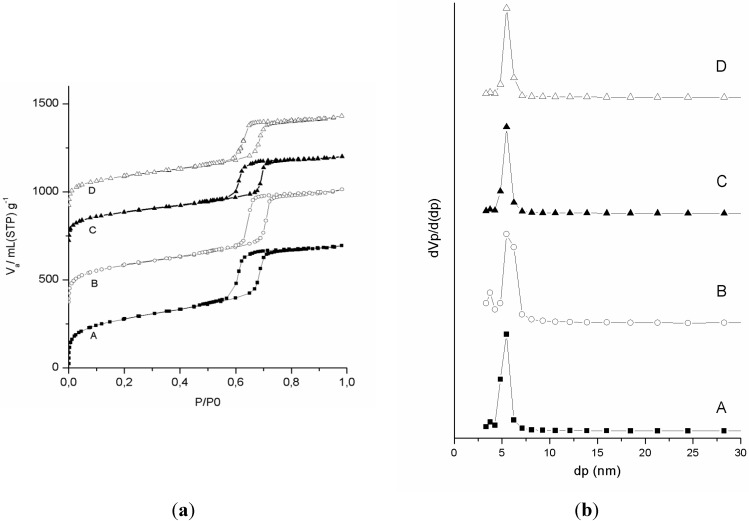
The nitrogen adsorption and desorption isotherms (**a**) and pore size distributions (**b**) of (A) EP; (B) BEP; (C) EP–(CH_2_)_3_–SH and (D) EP–(CH_2_)_3_–SO_3_H. The isotherms of BEP, EP–(CH_2_)_3_–SH and EP–(CH_2_)_3_–SO_3_H are vertically offset for clarity by 350, 700 and 900 mL (STP) g^−1^ (Standard Temperature and Pressure), respectively.

Nitrogen sorption measurements were performed to examine the porosity of the different materials obtained by the reaction pathway shown in Figure 1 (EP, BEP, EP–(CH_2_)_3_–SH and EP–(CH_2_)_3_–SO_3_H). The nitrogen adsorption and desorption isotherms are shown in Figure 2. The type IV isotherms with the condensation step at relative pressures between 0.55 and 0.75 and the H1 hysteresis of the solids clearly indicate that the materials are mesoporous and possess cylindrical pores with a narrow pore size distribution.

A summary of the properties of these materials is shown in Table 1. The materials exhibit high specific surface areas (S_BET_) ranging from 850 to 523 m^2^ g^−1^ and large total pore volumes around 0.84 mL g^−1^. The S_BET_ decreases when the material is functionalized due to the decoration of the pore walls with the bromine and later on with the propylthiol functionality but also due to the overall weight gain of the functionalized materials. The pore diameter of all the materials lies in the range of 6 to 5 nm. Only a minor shift to smaller pore diameters and a slight broadening of the pore size distribution is observed (Figure 2 and Table 1). The structural characteristics of the commercially available resin Amberlyst-15 are also presented in Table 1 for comparison. This ethenylbenzenesulfonic acid polymer is a strong acid ion exchange resin with unordered macropores. The material is also prone to swelling.

**Table 1:** Overview of the structural characteristics of the materials compared in this study.

Sample	Path	S_BET_^ a^ (m^2^ g^−1^)	V_p_^ b^ (mL g^−1^)	d_p_^ c^ (nm)
EP	A	850	1.03	5.8
BEP	B	663	0.84	5.6
EP–(CH_2_)_3_–SH	C	523	0.59	5.3
EP–(CH_2_)_3_–SO_3_H	D	688	0.72	5.4
Amberlyst-15	–	50	–	300

Notes: ^a^ Surface area calculated via the Brunauer-Emmett-Teller (BET) model; ^b^ Total pore volume at P/P_0_ = 0.98; ^c^ Pore diameter calculated via the Barrett-Joyner-Halenda (BJH) plot.

**Figure 3:**
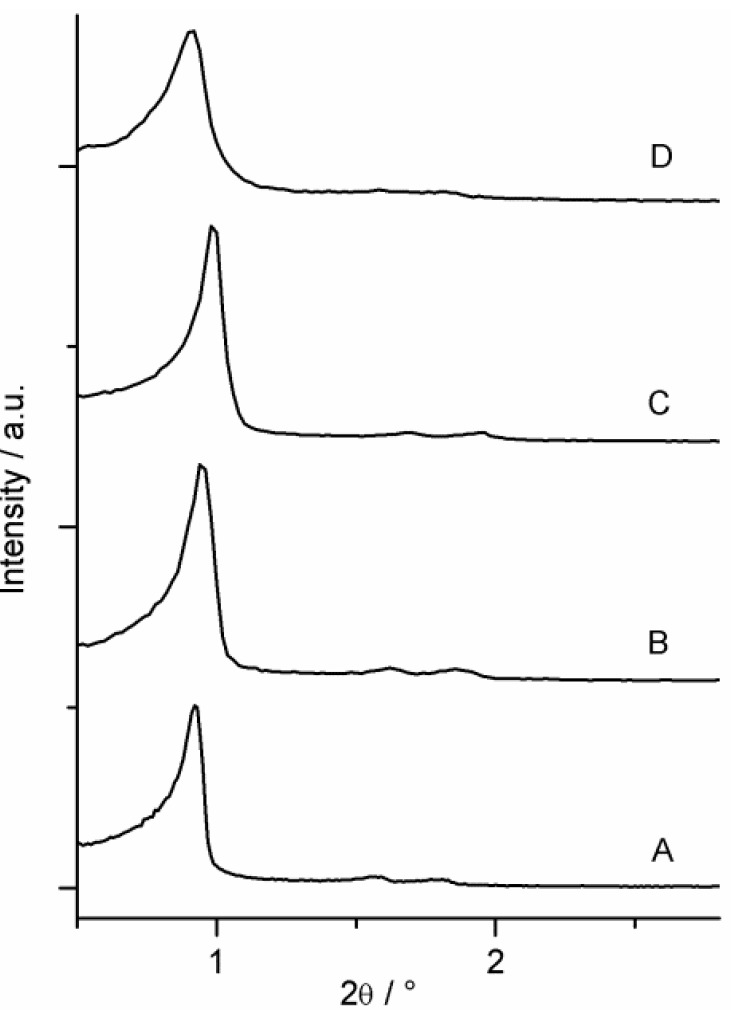
The powder X-ray diffraction patterns of (**A**) EP; (**B**) BEP; (**C**) EP–(CH_2_)_3_–SH; and (**D**) EP–(CH_2_)_3_–SO_3_H.

The XRD patterns of the materials in Figure 3 reveal three well-resolved signals originating from the low angle (100) and second-order (110) and (200) reflections. This evidently indicates that the materials possess a 2D-hexagonal ordered structure and thus retain their *P*6*mm* space group ordering throughout the syntheses. Only a slight broadening can be observed at the patterns of sample BEP, EP–(CH_2_)_3_–SH and EP–(CH_2_)_3_–SO_3_H.

It is quite remarkable that all the materials discussed in this study, show outstanding structural stability. The materials retain porosity and ordering after three consecutive reactions as can be seen from the nitrogen sorption and XRD data. These results also confirm the reported stability of Periodic Mesoporous Organosilicas [20,23].

Table 2 presents an overview of the chemical characterization of the solids after the different synthetic procedures. The bromination of the ethene bridge is a very straightforward reaction and approximately 25%–30% of the double bonds are brominated as the remaining fraction of double bonds are buried inside the walls and are unavailable for further reaction [45]. The subsequent substitution with the Grignard reagent results in thiol functionalities. The amount of thiol functionalities is determined using a silver titration [44,46]. After oxidation of the thiol groups using sulfuric acid and a thorough washing step, a total amount of 0.60 mmol H^+^ per gram of material has been observed. This also includes the intrinsic acidity of the PMO material originating from the surface silanols (~0.15 mmol g^−1^), as we described earlier [49]. It is clear that the conversion of the thiol containing PMO into the sulfonic acid containing-material has occurred via the oxidation process. This is also confirmed by Raman spectroscopy by the appearance of two signals in the region between 1160 and 1190 cm^−1^ (See figure S1 in supplementary information). Also, the thiol titration after oxidation showed a zero concentration of remaining thiol groups. Amberlyst-15 exhibits a high acidity of 4.7 mmol H^+^ g^−1^.

**Table 2:** Overview of the chemical characteristics of the materials compared in this study.

Sample	Functionality	mmol g^−1^
BEP	–Br ^a^	2.39
EP–(CH_2_)_3_–SH	–SH ^b^	0.40
EP–(CH_2_)_3_–SO_3_H	–SO_3_H ^c^	0.60 ^d^

Notes: ^a^ Determined gravimetrically; ^b^ Determined via silver titration; ^c^ Determined via acid/base titration; ^d^ The deviation between the amount of thiols and total acidity is due to the acidity of the surface silanols.

### 2.2. Catalytic Experiments and Recyclability

**Figure 4:**
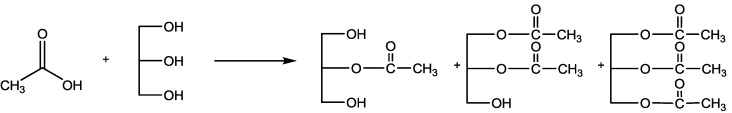
The esterification reaction: the acetylation of glycerol with the formation of glycerol monoacetate, glycerol diacetate and glycerol triacetate.

The catalytic ability of the sulfonic acid functionalized PMO material has been explored for an esterification reaction,* i.e.*, the glycerol acetylation reaction (Figure 4). The activity of EP–(CH_2_)_3_–SO_3_H is compared with a commercially available catalyst Amberlyst-15 and moreover the catalysts’ reusability is explored.

In this study the esterification of glycerol is probed due to its economic importance. Glycerol is an important by-product of first generation biodiesel and is produced in a relative large quantity [50]. This overproduction of glycerol can be used in order to develop second generation biodiesel which uses glycerol as a raw product. As carboxylic acid, acetic acid is probed as shown in the general reaction (Figure 4). Three products may in principle be obtained from this reaction: glycerol monoacetate (MAG), glycerol diacetate (DAG) and glycerol triacetate (TAG). However, experimentally, only MAG (~94%) and DAG (~6%) are formed using the specific catalytic conditions described in the experimental part [51].

**Figure 5:**
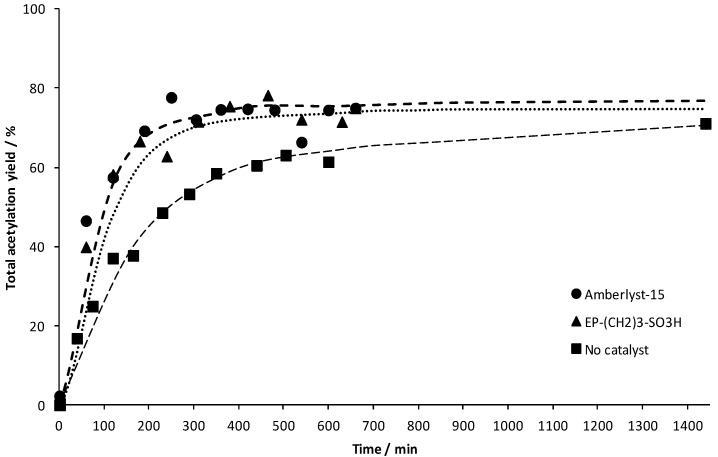
The total acetylation yield for the catalytic reaction with EP–(CH_2_)_3_–SO_3_H and Amberlyst-15. Also the blank reaction is represented for clarity. A catalyst loading of 0.25 g per 40 mL of glycerol was used. The lines are intended as visual aids only.

The catalytic activity of EP–(CH_2_)_3_–SO_3_H for the esterification of acetic acid with glycerol is presented in Figure 5 where the total acetylation yield is shown as a function of time. The total acetylation yield is defined according to the equation below:

(1) $$Yield_{\tau }(\%)=\frac{{\left[P\right]}_{t}}{{\left[HAC\right]}_{0}}\frac{\upsilon_{HAC}}{\upsilon_{p}}\times 100$$

where [*P*]_t_ and [*HAc*]_0_ represent the product and acetic acid concentration at a certain reaction time and at *t* = 0, respectively. Furthermore, *υ_HAc_* and *υ_P_* represent the stoichiometric coefficients of the acetic acid and the ester formed,* i.e.*, 1 for mono-substituted, 2 for di-substituted and 3 for fully substituted products, respectively. Also, as acetic acid contains acid protons which can induce a self-catalyzed process, the reaction in absence of any solid catalyst was monitored. Corresponding data are shown in Figure 5. It is clear that the sulfonated PMO possesses a significant catalytic activity with a yield of almost 80% for this reaction after ~300 min; whereas the blank test (without any solid catalyst involved) yielded only ~50% of esters after ~300 min. The conversion of Amberlyst-15 is shown in the same figure. Comparing the two materials clearly shows that EP–(CH_2_)_3_–SO_3_H exhibits a similar catalytic activity as Amberlyst-15, which is a well-performing catalyst in this type of reaction.

**Figure 6:**
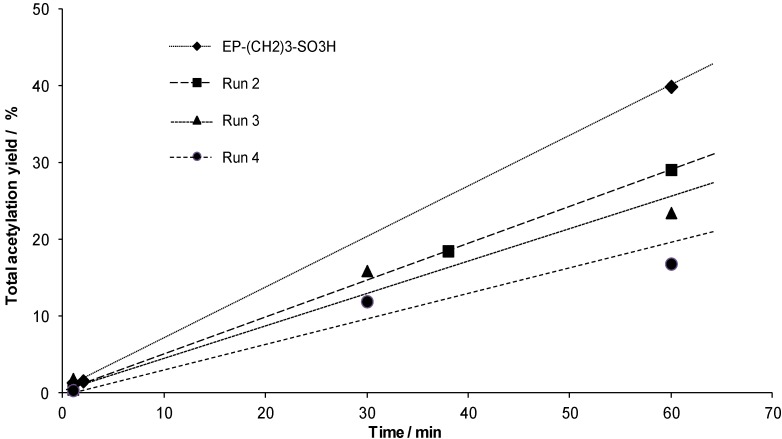
Recyclability experiments for EP–(CH_2_)_3_–SO_3_H with several runs during the first hour of the reaction. The lines are intended as visual aids only.

Furthermore, the recyclability of the sulfonic acid containing PMO material is studied for three consecutive runs. First, the initial rate of the catalytic reaction is studied for each run by focusing on the first hour of the acetylation (Figure 6). These experiments are all performed in the same catalytic set-up as the standard catalytic experiment. The solid is filtered after 1 hour and re-used without any further treatment in the subsequent run with a fresh reaction medium (run 2); this being performed again for two additional consecutive runs (runs 3 and 4). As one can see from the figure, the material still possesses catalytic activity for the acetylation after four runs. However, recycling of the material in the consecutive runs results in a slight decrease of the initial reaction rate.

**Figure 7:**
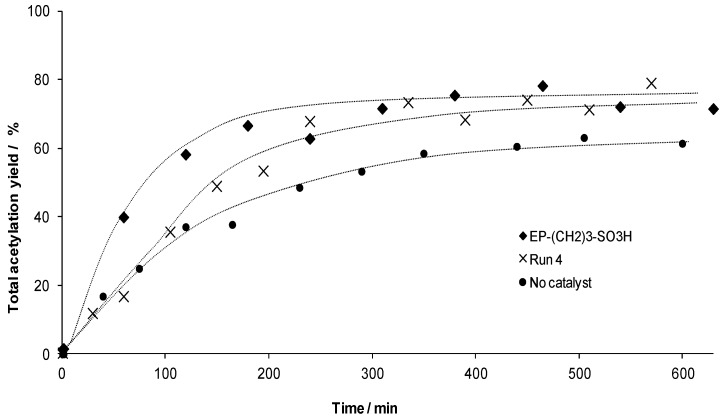
Recyclability experiment for EP–(CH_2_)_3_–SO_3_H: a comparison between the catalytic activity of the pristine material and the third catalytic run. The blank reaction is presented for clarity. The lines are intended as visual aids only.

After the last catalytic run,* i.e.*, run 4, the total acetylation yield is monitored for approximately 10 h in order to compare it with the acetylation yield of the pristine sulfonated PMO material, EP–(CH_2_)_3_–SO_3_H (Figure 7). Although a decrease in the initial reaction rate was observed as already mentioned, at the third consecutive run, the material still reaches the equilibrium after approximately 5 h and finally results in the same acetylation level as the fresh pristine material.

**Figure 8:**
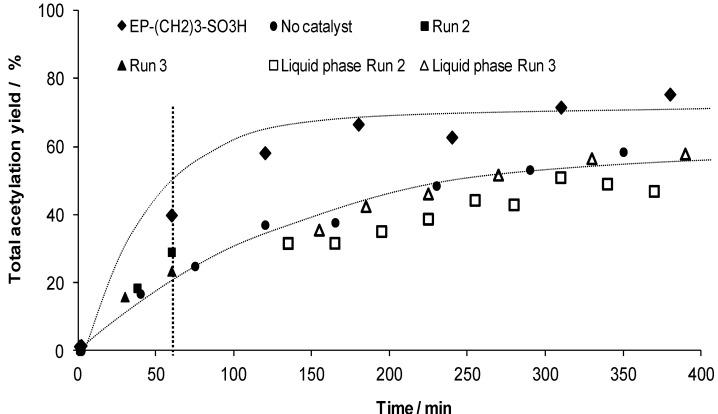
The total acetylation yield for the EP–(CH_2_)_3_–SO_3_H, the second and third run. After 60 min (represented by the vertical line) the liquid is separated from the catalyst and the catalytic activity of the liquid phase of run 2 and 3 is further followed in function of time (open square and triangle). The blank reaction is presented for clarity. The dotted lines are intended as visual aids only.

Moreover, additional tests are performed to evaluate the actual heterogeneous character of the observed catalytic activity. Therefore, the solids are removed from the liquid after 1 h along the first and second runs, and the corresponding recovered solutions are kept under the catalytic conditions to follow the occurrence of a further evolution of the total acetylation yield without solid catalyst in the system anymore. As can be seen from Figure 8, it is clear that the acetylation occurs much slower,* i.e.*, in the range of the blank, when the catalysts are removed of the reaction media, than when the catalyst is maintained in the reactor for the whole test duration.

## 3. Experimental Section

### 3.1. Materials

The following reagents were used for the synthesis and characterization: Grubbs’ first generation catalyst (PCy_3_)Cl_2_Ru=CH–Ph, pluronic P123 (PEO_20_PPO_70_PEO_20_), 1-butanol, bromine, 3-chloro-1-propanethiol (98%), magnesium turnings (98%), sodium chloride, potassium thiocyanate, ammonium iron sulfate dodecahydrate, nitric acid (65%), iron (III) chloride (98%; anhydrous), toluene (anhydrous), acetonitrile, sulfuric acid (H_2_SO_4_), silver nitrate were obtained from Sigma-Aldrich (Bornem, Belgium). Acetone (>99.5%) was acquired from VWR (Belgium). Tetrahydrofurane (rotidry) and hydrochloric acid (37%; p.a.) were purchased at Carl Roth (Karlsruhe, Germany). Vinyltriethoxysilane (97%) was acquired from ABCR (Karlsruhe, Germany). The following reagents were used for the catalytic reaction: acetic acid (puriss. p.a., ACS reagens, ≥99.8%, GC/T), glycerol (puriss. p.a., ACS reagens, anhydrous, dist., ≥99.5%) were purchased from Fluka (Bornem, Belgium). 1-Propanol, 1-heptanol (98%) and o-xylene (98%) were obtained from Sigma Aldrich. All chemicals were used as received.

### 3.2. Synthesis of Pure E-1,2-Bis(Triethoxysilyl)Ethene (E-BTSE)

The diastereoisomerically pure E-isomer of the ethenylene precursor was synthesized according to a procedure described by our research group [43,45]. An amount of 0.0535 g of the Grubbs’ first generation catalyst and 42.95 mL of vinyltriethoxysilane (VTES) were mixed together under inert atmosphere. The mixture was stirred for 1 h at room temperature and subsequently refluxed. After 3 h, a distillation was performed to remove remaining VTES. Afterwards, the colorless E-1,2-bis(triethoxysilyl)ethene (E-BTSE) was distilled off under vacuum (~0.1 Pa).

### 3.3. Synthesis of Ethenylene Bridged Periodic Mesoporous Organosilica (Figure 1; Pathway A)

In a typical synthesis, reported by our group [45], a total amount of 1.00 g of P123 was placed in a flask and 47.80 mL of distilled H_2_O, 3.42 mL concentrated HCl and 2.45 mL 1-butanol were added. The solution was stirred for 1.5 h at room temperature and subsequently 1.86 mL of E-BTSE was added to the mixture. Next, the mixture was vigorously stirred at 35 °C. After 4 h, the temperature was raised to 90 °C and aged for 16 h. Afterwards, the mixture was left to cool down, and the white solid was filtered and washed with acetone and distilled water. Finally, P123 was removed via Soxhlet extraction with acetone for 5 h. The white solid, denoted as EP, was dried at 120 °C under vacuum (~0.1 Pa).

### 3.4. Synthesis of BEP and EP-Propylthiol (Figure 1; Pathways B and C)

The synthesis of this material was extensively described by our research group [38,46]. A certain amount of EP was first brominated with bromine gas under vacuum for 3 h. The resulting material was then dried at 90 °C for 16 h. Magnesium (0.74 g), iron(III) chloride (0.54 g) and dry tetrahydrofuran (THF) (30 mL) were mixed under inert atmosphere and stirred for 30 min at 50 °C. Next, 3-chloro-1-propanethiol (0.22 mL) was added and left to stir for 4 h at room temperature. Subsequently, the solution was added to 0.70 g of dry brominated EP. The resulting mixture was stirred for an additional 24 h at 40 °C. Then, the solid was filtered and washed several times with THF, 2 mol L^−1^ HCl, H_2_O and acetone. Finally, the material was dried at 90 °C for 16 h under vacuum (~0.1 Pa) and denoted as EP–(CH_2_)_3_–SH.

### 3.5. Synthesis of EP-Propylsulfonic Acid (Figure 1; Pathway D)

A volume of 25 mL H_2_SO_4_ (2.5 mol L^−1^) was added to 0.50 g of EP–(CH_2_)_3_–SH and stirred at room temperature. After 1 h, the solids were filtered and washed thoroughly with water and acetone. Finally, the material was dried at 90 °C for 16 h under vacuum (~0.1 Pa) and referred to as EP–(CH_2_)_3_–SO_3_H.

### 3.6. Determination of the Amount of Reachable Thiols

A titration is performed as described in the literature [44]. A solution of silver nitrate with a known concentration is added to 0.050 g of material and left to stir until the equilibrium is reached. The excess of silver is titrated with potassium thiocyanate and FeNH_4_(SO_4_)_2_·12H_2_O in 0.3 mol L^−1^ HNO_3_ as indicator.

### 3.7. Determination of the Total Acidity

The material containing sulfonic acid groups was stirred for 24 h in 20 g of 2 mol L^−1^ NaCl. Next, an acid-base titration was achieved with NaOH and phenolphtaleine as indicator.

### 3.8. Esterification of Acetic Acid with Glycerol

The catalytic reaction was carried out at 105 °C and at atmospheric pressure in a round bottom flask reactor equipped with a magnetic stirrer. The sulfonic acid containing material was dried prior to use at 105 °C under vacuum (~1.33 Pa) for 18 h. The following concentrations of the reagents were used: 100 g acetic acid/L of glycerol and the catalyst concentration was 6.25 g/L of glycerol. First, the catalyst and glycerol are added together to the flask and are brought to reaction temperature. When the temperature is reached, the acetic acid is injected. Before analysis the samples are extracted with 1-heptanol and using *o*-xylene as an internal standard. The total yield of the esters was determined with gas chromatography. After one minute a sample of the reaction mixture is taken and analyzed with gas chromatography. This sample represents the starting point of the catalytic experiment. The recyclability experiments were carried out under identical conditions as the catalytic tests. Therefore, the catalyst was separated from the liquid phase after 1 h and reused in a consecutive run without further treatment. The liquid phase was kept under reaction conditions and further followed as a function of time. Gas chromatography was performed with a Varian CP-3800 using a Cp-Sil 8CD column (Varian, Palo Alto, CA, USA) with a FID detector.

### 3.9. Characterization

Nitrogen sorption measurements were carried out by a Belsorp-mini II gas analyzer at 77 K. The specific surface area (S_BET_) was determined by the BET equation (p/p_0_ = 0.05–0.15). The pore size distribution was determined from the desorption branch of the isotherm using the Barrett-Joyner-Halenda (BJH) theory. The samples were pretreated at 90 °C while degassing (~0.1 Pa). X-ray diffraction (XRD) measurements were conducted with an ARL X’tra X-ray diffractometer of Thermo Scientific (Waltham, MA, USA) equipped with a Cu K_α1_ tube and a Peltier cooled lithium drifted silicon solid stage detector. Raman spectra are recorded using a Raman type FRA106/S spectrometer of Bruker (Karlsruhe, Germany), equipped with a Nd-YAG laser (λ = 1064 nm).

## 4. Conclusions

Periodic Mesoporous Organosilica functionalized with sulfonic acid groups has been successfully synthesized and characterized. An ethenylene bridged PMO material was chosen as starting material and this was further post-modified in several steps. A bromination and subsequent substitution reaction was used followed by an oxidation turning the material into a solid acid catalyst. This material has been investigated for the acetylation of glycerol. The material showed an equal activity as Amberlyst-15; the latter being usually considered as the reference most-efficient catalyst material for such kind of reactions. Recyclability experiments showed that the sulfonated Periodic Mesoporous Organosilica is reusable and showed the same total acetylation yield after three runs. Homogeneous tests showed that the acetylation of glycerol indeed occurs heterogeneously, suggesting the absence of leaching, e.g., of sulfonate species, in the medium, and thus the high stability of our material under working conditions.

## Supplementary Files

Supplementary File 1
