# Supplementary material for: Periodic Mesoporous Organosilica Functionalized with Sulfonic Acid Groups as Acid Catalyst for Glycerol Acetylation
Source: Materials (Basel). 2013 Aug 16;6(8):3556–70. doi: 10.3390/ma6083556 (PMC5521322; doi:10.3390/ma6083556)
Supplement: Supplementary File 1 [file materials-06-03556-s001.pdf]

Materials **2013**, *6*, 3556-3570; doi:10.3390/ma6083556

OPEN ACCESS

materials

ISSN 1996-1944

www.mdpi.com/journal/materials

Article

Supporting Information

Figure S1. Raman spectrum of EP and EP–$(CH_2)_3$–SO_3H. The highlighted area shows the signals of the sulfonic acid group.

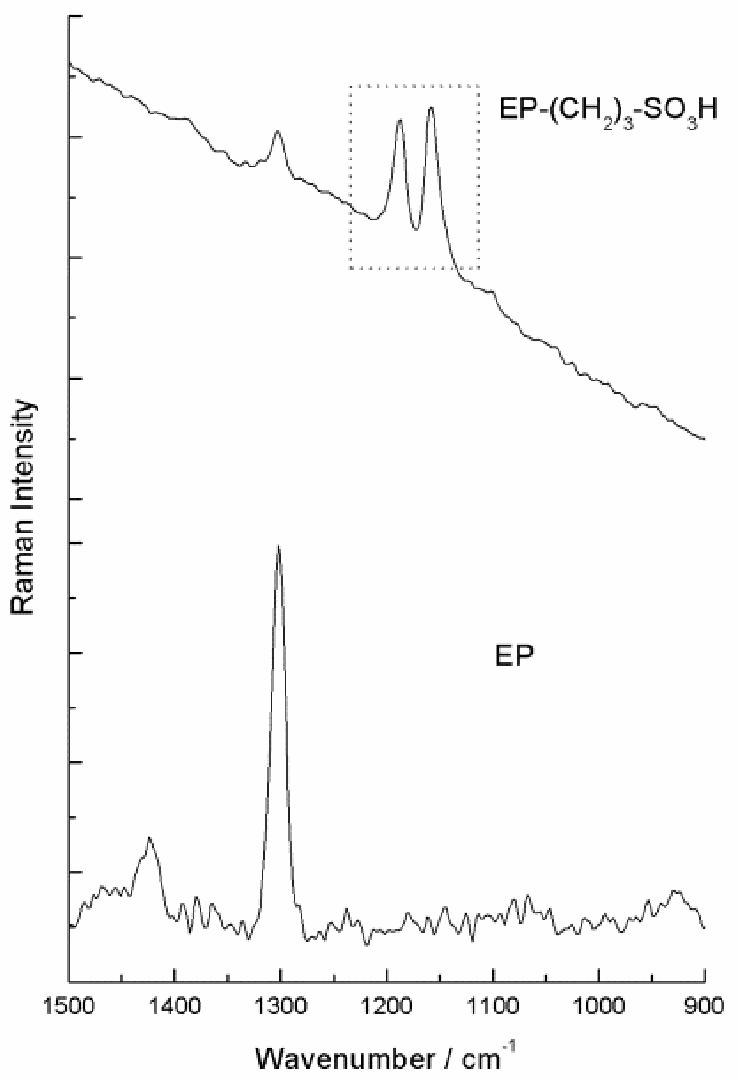

© 2013 by the authors; licensee MDPI, Basel, Switzerland. This article is an open access article distributed under the terms and conditions of the Creative Commons Attribution license (http://creativecommons.org/licenses/by/3.0/).
